# Self-assembled ordered structures in thin films of HAT5 discotic liquid crystal

**DOI:** 10.3762/bjoc.6.51

**Published:** 2010-05-20

**Authors:** Piero Morales, Jan Lagerwall, Paolo Vacca, Sabine Laschat, Giusy Scalia

**Affiliations:** 1 ENEA, C. R. Casaccia, Via Anguillarese 301, I-00060 S. Maria di Galeria (Roma), Italy; 2 Institute of Chemistry – Physical Chemistry, Martin-Luther-University Halle-Wittenberg, Von-Danckelmannplatz 4, D-06120 Halle, Germany; 3 ENEA, C. R. Portici, P.le E. Fermi, I-80055 Portici (Naples) Italy; 4 Institute of Organic Chemistry, University of Stuttgart, Pfaffenwaldring 55, D-70569 Stuttgart, Germany

**Keywords:** AFM, discotic liquid crystals, hexapentyloxytriphenylene, self-organization, thin films

## Abstract

Thin films of the discotic liquid crystal hexapentyloxytriphenylene (HAT5), prepared from solution via casting or spin-coating, were investigated by atomic force microscopy and polarizing optical microscopy, revealing large-scale ordered structures substantially different from those typically observed in standard samples of the same material. Thin and very long fibrils of planar-aligned liquid crystal were found, possibly formed as a result of an intermediate lyotropic nematic state arising during the solvent evaporation process. Moreover, in sufficiently thin films the crystallization seems to be suppressed, extending the uniform order of the liquid crystal phase down to room temperature. This should be compared to the bulk situation, where the same material crystallizes into a polymorphic structure at 68 °C.

## Introduction

Discotic liquid crystals are an interesting type of organic semiconductors that allow long-range charge transport thanks to the spontaneous formation of channels as a result of molecular self-organization into columns and the orbital overlap of neighboring molecules [1–2]. In particular, columnar phases are very appealing for applications since the transport of charges occurs along the columns, which then behave like molecular wires. Their good performance makes them promising for use as transistors [3] in electronic circuits, for light emitting diodes [4] and in solar cells [5–6]. Liquid crystals (LCs) possess a long range, even if imperfect, order that is beneficial for creating a macroscopic common molecular orientation which can be potentially controlled, thus making it possible to choose the direction of charge flow. Moreover, the degree of molecular order affects the charge transport efficiency, which improves with increasing order.

Efforts to improve the electronic performance of such systems have focused not only on the synthesis of optimized molecules but also on the aligning methods. In fact while rod-like (calamitic) LCs can be easily aligned along chosen directions by the use of treated surfaces or electric or magnetic fields, these treatments are almost always ineffective for discotic LCs. Without a macroscopic, uniform alignment the excellent properties of discotic LCs cannot be expressed at their best. Moreover, the ability to select the direction of the column alignment is also desirable since the optimum direction depends on the application [7]. Use of discotics as transistors requires LC columns bridging the electrodes, usually parallel to the substrates. This corresponds to the so-called planar alignment with the molecular planes perpendicular to the substrate and the columnar axis parallel to it. On the other hand, for solar cells or OLEDs the columns should be perpendicular to the electrode coated substrate, in what is referred to as homeotropic alignment, with the molecules lying flat on the substrate surface. While some progress has been made in identifying alignment methods, these are not generally effective for all types of discotics, with some materials aligning as expected and others not. Methods to align discotics include the use of treated surfaces [8–9], the Langmuir–Blodgett technique [10], zone-casting processes [11] or the use of magnetic fields [12]. With the above methods planar alignment is mostly achieved and often fibrillar structures are observed. Uniform homeotropic alignment in thin films is rarer and often difficult to achieve. The properties of the discotic thin films are also important for applications since the uniformity of the alignment on macroscopic scale and the stability of the alignment with the temperature are fundamental for the actual implementation of discotics in devices.

**Scheme 1:**
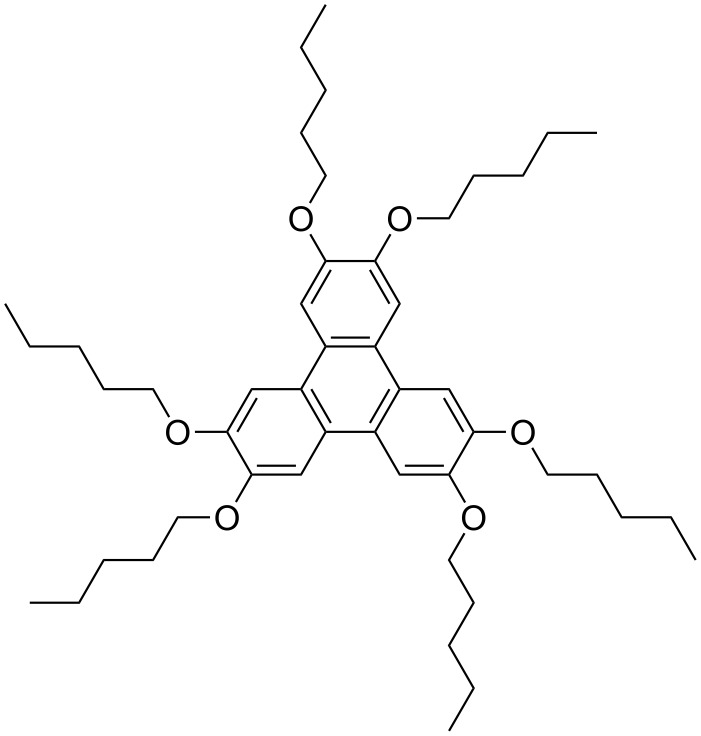
Hexapentyloxytriphenylene (HAT5).

In the present work we study thin films of hexapentyloxytriphenylene, known as HAT5 for short (Scheme 1), a historically important discotic molecule which allowed the proof of photoconductivity in discotic LCs [13]. Besides exhibiting high charge mobility this material is, unlike many other discotic LCs, relatively easy to align uniformly in a homeotropic configuration between two glass plates, thus allowing time-of-flight measurements of photoconductivity. While most of the studies are with such geometry, which is obtained by confining the LC between indium tin oxide (ITO)-coated glass plates at micrometer distance, very little is known about the organization of HAT5 in films of submicrometer thickness and their characteristics. Such films, produced by drop-casting and spin-coating, are the focus of this study.

## Results and Discussion

**Figure 1:**
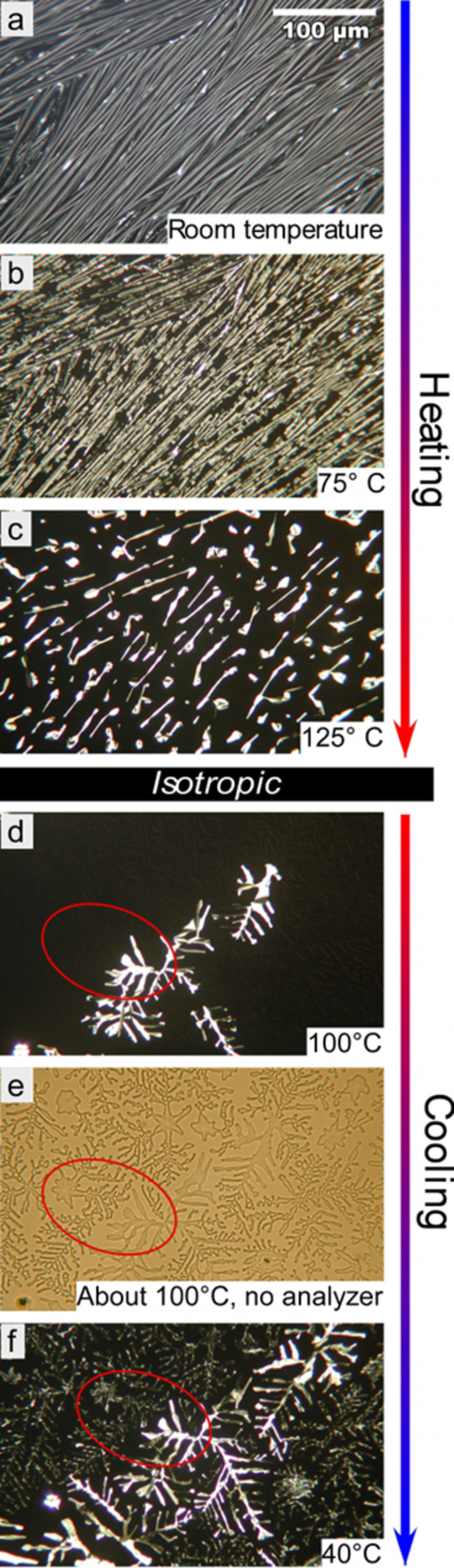
Optical microscopy textures of a film prepared by drop-casting on heating to the isotropic phase and subsequent cooling. All images were taken with crossed polarizers, except (e) which was taken with the analyzer removed.

Different preparation techniques obviously produce films of different thicknesses and uniformity; drop-casting yields thicker and less uniform samples as compared to those prepared by spin-coating. The former were thick enough to show birefringence, thus making them suitable for optical microscopy investigations. As can be seen in Figure 1a, the sample has a striped texture, which is uncharacteristic of columnar phases studied in standard sample cells (typical thickness of several microns), with long fibers aligned locally along a common direction. The alignment of the director (the average orientation of the main molecular symmetry axis) is planar, as evidenced by the non-zero birefringence, with the column axes parallel to the substrate. The film was heated to study the phase sequence, which revealed that the temperatures of the transitions were somewhat shifted from those of bulk samples (crystal 69 °C Col_h_ 122 °C isotropic). A first change in texture was observed around 75 °C (Figure 1b), corresponding to melting into the columnar liquid crystalline phase (Col_h_). The new texture retained the striped character but broke up into many small domains. At 125 °C (Figure 1c) the disappearance of birefringence revealed the transition to the isotropic phase.

The textures were also monitored on cooling and showed the liquid crystal phase formation at about 110 °C with the texture now typical of a discotic columnar phase with the characteristic bright dendritic features in a black background (Figure 1d). The area in the red oval includes bright and dark regions in the polarizing microscopy image. Observing the same area without the analyzer (Figure 1e) revealed a different, non-birefringent dendritic regime, suggesting a homeotropic LC alignment. At crystallization (Figure 1f), the alignment within each regime was partially lost, resulting in the previously non-birefringent homeotropic areas becoming visible between crossed polarizers.

The striking difference between the initial and final texture after the thermal treatment demonstrates that the starting arrangement must have been strongly influenced by the preparation process, the flow direction of the solution and the assembly during solvent evaporation probably being critical for the development of the fibrillar structure (we will propose a more detailed explanation below). However, this alignment is apparently not stable in this relatively thick film and when the LC is heated into the isotropic phase this organization is lost. On subsequent cooling into the columnar phase this instead forms according to the interaction with the surface, resulting in a standard homeotropic alignment configuration typical of bulk samples.

**Figure 2:**
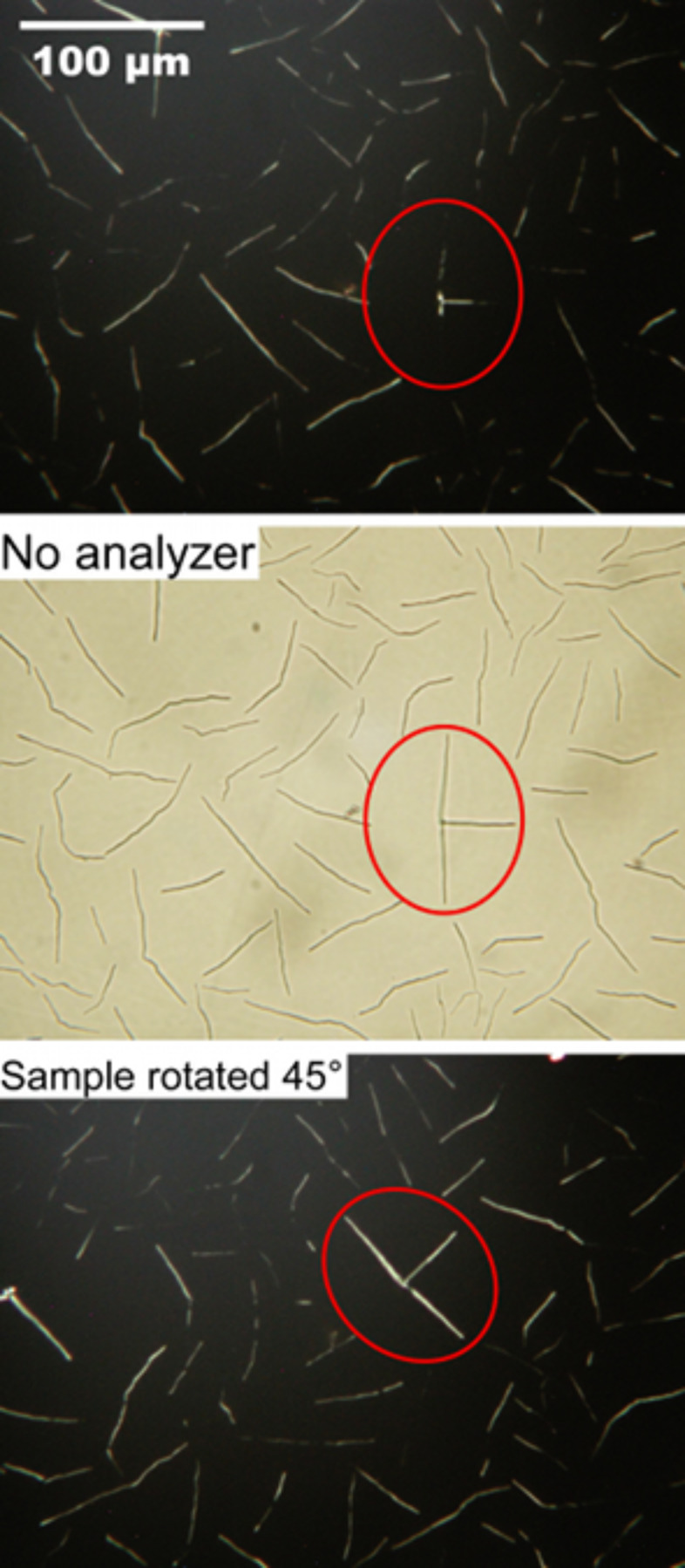
Optical microscopy images of a spin-coated sample with linear structures thick enough to be detected optically. Since their birefringence depends on the relative orientation of the optical axis and the polarizers, we have used a reference area, enclosed in the red oval, for demonstrating the uniform, planar alignment of the director in the linear structures. The upper and lower images were obtained with crossed polarizers while the middle one was taken without the analyzer to show the complete sample morphology.

**Figure 3:**
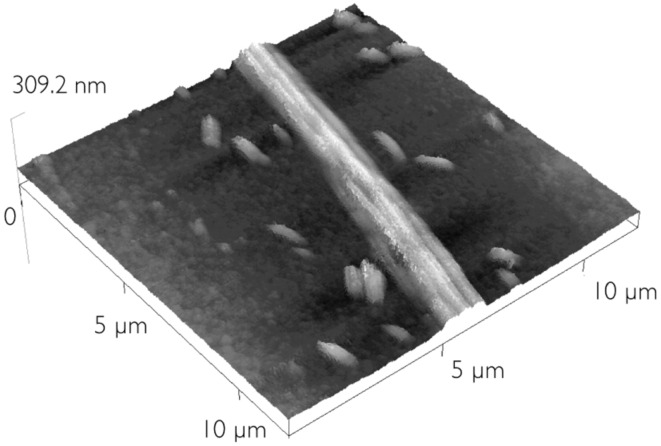
AFM scan of a thick rope, of similar size to the linear structures observed by optical microscopy. AFM shows that they are actually composed of several thinner fibers.

The samples prepared by spin-coating resulted in a thinner layer of material as suggested by a basically black appearance in the polarizing microscope. In some cases some birefringent lines with planar alignment, with widths in the range 2–3 µm, could be detected (Figure 2). Other samples prepared with settings that ensured thinner films had a uniformly black aspect. The origin of the black background is the extremely small thickness of the LC film, giving negligible effective birefringence, even for a director in planar orientation. The analysis of these regions thus relied mainly on atomic force microscopy (AFM, Figure 3–Figure 9). This technique confirmed that the black background observed by optical microscopy is not simply the uncovered substrate but it shows a fibrillar structure reminiscent of the optical microscopy textures from the thicker films produced by casting.

It is thus reasonable to deduce that the LC is also aligned planarly in these thin films. Indeed, the linear structures that could also be detected optically in the spin-coated films were birefringent with a well-defined optical axis, such that they appeared and disappeared upon rotation of the sample; an example is shown in the red ellipse in the top and bottom pictures of Figure 2. It was possible to localize these structures by AFM as shown in Figure 3. Here an example with overall diameter in the order of 1.5–2 μm (similar to the lines detected optically) is shown, together with small elongated aggregates. The substrate roughness, evaluated as the root mean square of the AFM topographic values of representative areas of the image is 1.6 nm.

**Figure 4:**
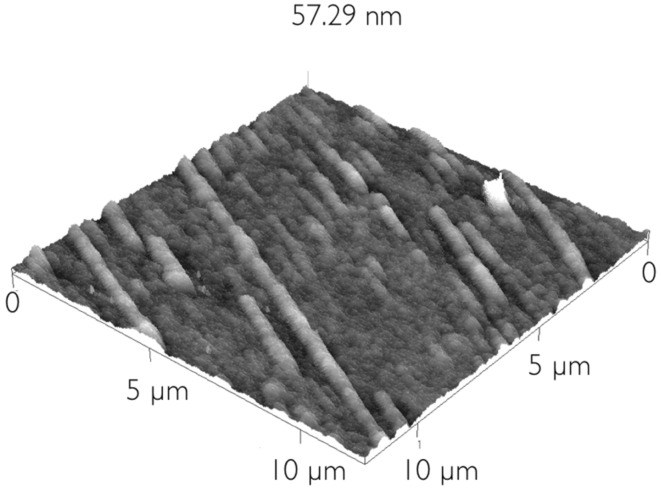
Thinner, isolated fibers, similar to the ones constituting the larger rope in Figure 3 are present in other areas of the sample.

The structure actually appears to be a kind of rope, formed by thinner fibers, each of diameter in the order of 0.5 μm. In other regions it was possible to observe the thinner fibers, of dimensions comparable to the ones constituting the thicker rope, not in aggregates but lying separately on the substrate as can be seen in Figure 4. These thinner fibers can also be very long, at least tens of micrometers.

The phase transition behavior in the thin spin-coated films could be studied in the thick linear structures as these were observable by optical microscopy. Interestingly, this revealed a drastic difference from the drop-cast sample. In the ropes of the spin-coated film no phase transition was detected upon heating from room temperature up to 129 °C, when the material became isotropic. Therefore only one transition was visible, presumably the liquid crystalline–isotropic transition, demonstrating a dramatic stabilization of the LC order over a much larger temperature range than in bulk samples. While we cannot rule out that the sample is in a glassy state at low temperatures, the important observation is that the large-scale order is retained. The increase in the temperature range of the ordered state is an effect of the confinement of the molecules into a very thin structure, analogous to the observations made in liquid crystal confined in electrospun polymeric microtubules [14]. Moreover, the spatial confinement gives also stabilization of the alignment. Indeed, after heating the spin-coated sample to the isotropic phase and then cooling it to the LC state, the original alignment was preserved and the texture appeared unchanged from the initial state, in contrast to the observations made on the thicker drop-cast samples. Thus, these two different preparation methods lead to a great difference in the stability of molecular alignment and in the temperature behavior of the material, even starting from the same solution. This behavior is important for applications since it may allow large-scale uniform alignment over a substantially wider temperature range compared to the bulk LC.

**Figure 5:**
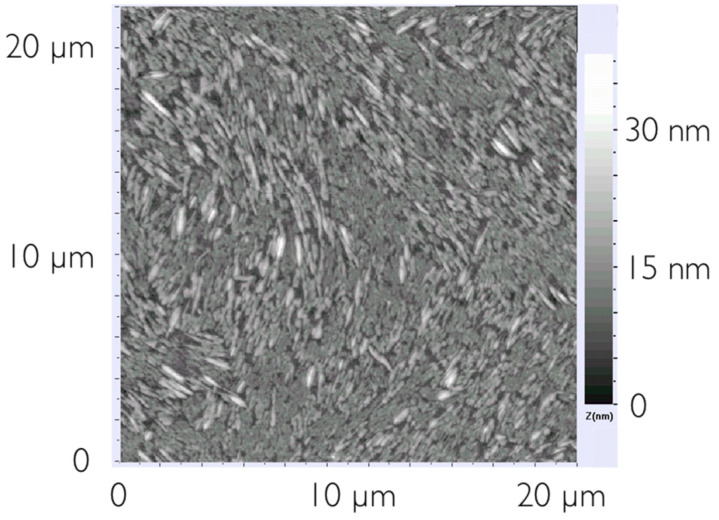
Small, elongated aggregates form a nematic-like texture, possibly due to the formation of a lyonematic phase during solvent evaporation.

Because of the close resemblance between the fibers in the ropes, visible by optical microscopy due to their birefringence, and the non-aggregated and optically invisible fibers observable only by AFM, we can infer that the alignment in the latter is also planar. Moreover, if the stabilization of the planar alignment occurs in the larger structures, the effect ought to be even more enhanced in smaller arrangements. Besides these fibers that can be extremely long, areas with densely packed “elongated droplets” can also be found, as shown in Figure 5. These have a width in the order of hundreds of nanometers while the length is in the micron range. Moreover, they are locally ordered along the same direction, but over the sample area this direction changes smoothly, producing a pattern strongly reminiscent of nematic organization.

One explanation for the formation of planar alignment in thin films (generally thicker than those studied here, however) has been proposed by Grelet and Bock [15] in terms of the difference in surface tension at the different interfaces. In that case the stable LC order in the films appears only upon thermal annealing. While the same process might be occurring in our system, the evidence of nematic order – absent in the phase sequence of HAT5 – is difficult to explain following this scenario, suggesting that another or additional mechanism plays a role. One possible explanation could be that, as the solvent evaporates a concentration is reached at which aggregates of columnar stacks exhibit an organized phase of lyomesomorphic type, like the soft columnar nematic phases of diluted discotics described in other works [16–18]. Discotic molecules aggregate in small columnar stacks in dodecane and at a certain concentration these stacks behave as molecules in a calamitic nematic phase, aligning accordingly. In reference [19] it is reported that aggregation of the discotic molecules already starts when they are in solution. This assembly can remain or influence the final structure together with processing conditions. When all the solvent has evaporated, the organization into molecular stacks and their super-organization in nematic order may be frozen in, explaining the peculiar texture shown by AFM (Figure 5) as well as the non-standard initial textures of the drop-cast sample observed by optical microscopy. The tendency to form organized structures even on the small scale is striking, and it is clearly important to understand how to control and tune a desired assembly.

**Figure 6:**
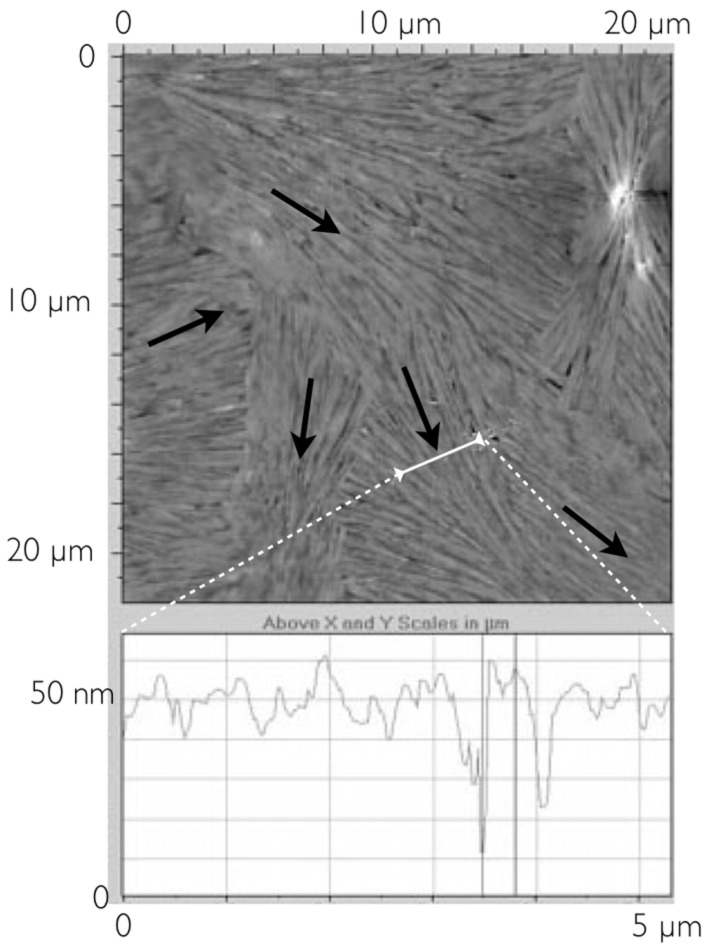
AFM image of a spin-coated sample from a solution of 6 mg/ml. Below the surface profile along the white arrow shows a presumable fiber layer thickness of about 40 nm.

We therefore carried out investigations to study the impact of the change in the concentration of HAT5 in toluene at the same settings for the deposition on substrates of equal size via spin-coating. Three concentrations were examined: 6 mg/ml, 3 mg/ml and 1.5 mg/ml. The fibrillar texture appeared in all cases, rendering the assessment of the film thickness more difficult due to the uneven surface. We found that for the highest concentration, 6 mg/ml, the layer thickness ranges between 30 and 50 nm. An example of the surface roughness is shown in the lower part of Figure 6. The surface is characterized by fiber-like structures that are locally aligned although the alignment changes in different domains. Arrows have been drawn as a guide to the eye to identify the local alignment. Unlike the arrangement of Figure 4, no “particle-like” units could be identified but only rather long fibers that smoothly bend together with neighboring fibers. It is not easy to identify the individual fibers and quantify the dimensions because they are closely packed together. They appear quite thick even although the section of the surface given by the AFM identifies also some thinner features, suggesting that the thick structures may be thin fibers aggregating into superstructures.

**Figure 7:**
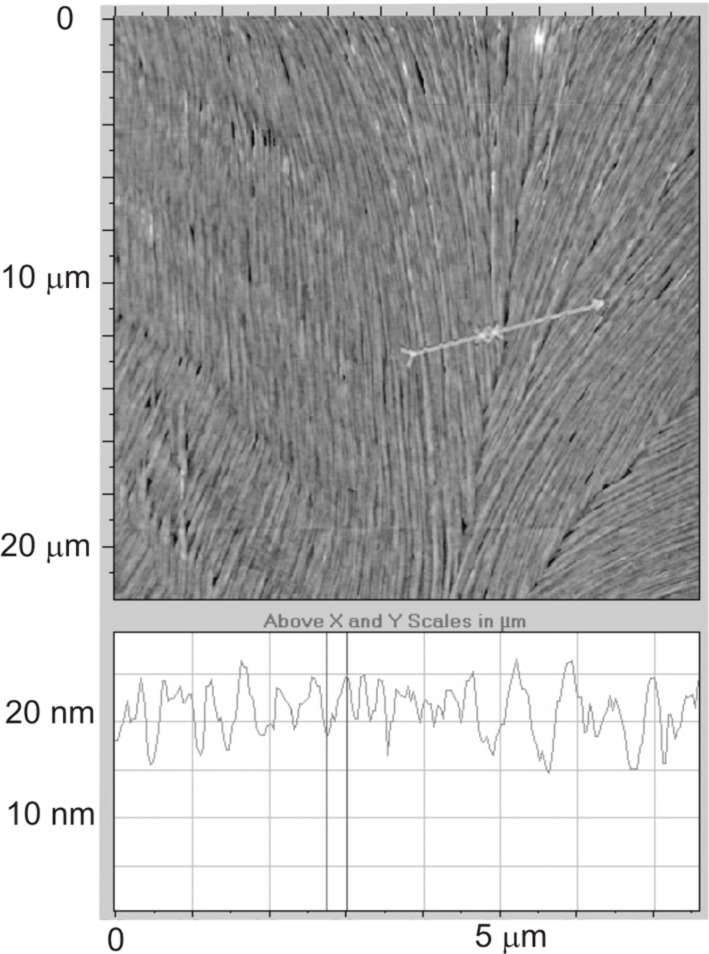
AFM image of the sample prepared from a concentration of 3 mg/ml.

As expected reducing the concentration decreases the film thickness to values in the range of 10–20 nm for the sample produced from the 3 mg/ml solution, cf. Figure 7. Interestingly, the fibrils now seem to align easier with each other, forming larger areas of uniform alignment compared to the more concentrated samples. While it is apparent that the area of uniform alignment is larger than in the thicker sample, the most striking feature is the thinning of the fibers that appear more distinct and almost continuous from the top of the scan to the bottom thus indicating a length of at least 20 μm.

**Figure 8:**
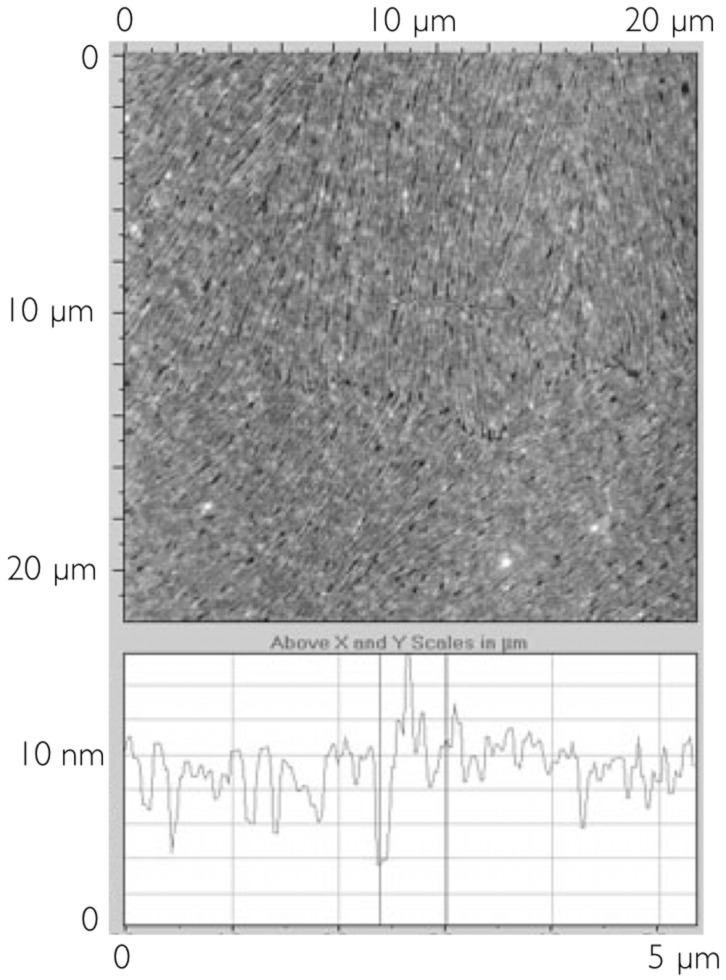
Surface topography of the sample prepared from a concentration of 1.5 mg/ml visualized by AFM. The diagram below shows a section of the surface.

For the even smaller concentration of 1.5 mg/ml, the resulting film thickness ranges between 5 and 10 nm. The AFM investigation of Figure 8 shows a structure of quite thin fibers still largely with a common orientation. Measurement of the lateral fibril dimension here is difficult, but they appear thinner that those in the previous samples, and the surface profile shows more subtle features.

**Figure 9:**
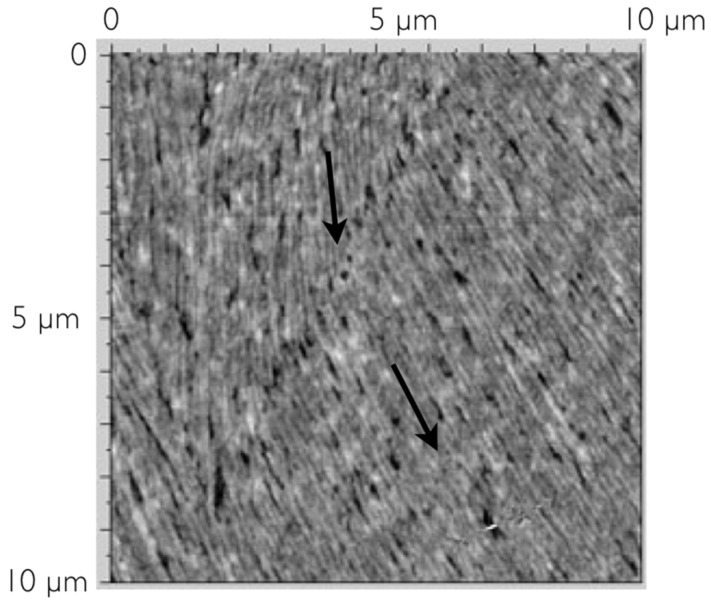
Higher resolution scans of the fiber structure of the low concentration sample.

In Figure 9, a higher resolution image of the fiber structure for this lower concentration sample is shown. The width of these fibers appears to be in the order of hundreds of nanometers and their self-assembly renders small parts of the substrate visible. In the region shown, the alignment of the group of fibers changes abruptly by about 20° (the two arrows show the local director orientations). At the change point the fibers break, giving rise to what looks like a long trench from the top centre to the lower left-hand corner.

An overview of the results obtained for cast and spin-coated samples, in comparison with bulk samples, is provided in Table 1.

**Table 1:** Overview of results for the different films compared to bulk samples.

Sample	Bulk	Cast	Spin-coated

Thickness	Typically a few µm	Enough for optical birefringence	5–50 nm
Phase sequence	crystal 69 °C Col_h_ 122 °C isotropic	crystal 75 °C Col_h_ 125 °C isotropic	glass (?) 129 °C isotropic
Typical alignment and structure on ITO glass slides	Homeotropic, columnar	Planar and homeotropic, (metastable) fiber-like structure	Planar, stable fibers and ropes

## Conclusion

The discotic liquid crystal HAT5 showed the tendency to form fiber-like structures in films prepared from solution using different methods. In the thicker samples prepared by drop-casting this texture is however not stable, heating to the isotropic phase and subsequent cooling resulted in the typical alignment of HAT5 known from sandwich cells. For all thinner samples, prepared by spin-coating, a fiber-like texture was observed, persistent after thermal treatment and stable over a larger temperature range. The most common arrangement observed is one of very long fibers that appear thinner at the lower concentrations of the starting solution, resulting in generally thinner films. Further studies employing techniques such as X-ray scattering are needed for the elucidation of the definite molecular arrangements. However, the preference to form long fiber structures and the columnar hexagonal phase possessed by HAT5 in bulk suggest that this phase may be present in the observed fibers.

We conclude that even a simple technique such as spin-coating allows organization of discotic liquid crystals in micrometer-sized regions of uniform planar alignment. The self-organizing nature of discotics is thus a powerful and promising tool for creating controlled nanometric or micrometric structures.

## Experimental

HAT5 was dissolved in toluene at different concentrations and deposited on square glass substrates of 2.5 cm side length, coated with indium tin oxide (ITO). The deposition of the solution was performed by drop-casting or spin-coating, the latter at a speed of 500 rpm for 10 s. A starting concentration of 7.2 mg/ml was initially chosen to ensure a macroscopic coverage of the substrate surface. All the samples were then kept for 2 h at 56 °C in a vacuum oven to ensure complete evaporation of the solvent.

The investigations of the deposited layers were performed by polarizing optical microscopy and by atomic force microscopy. An Olympus BH-2 optical microscope equipped with an Instec temperature controller allowed the characterization of the phase transitions and the assessment of the orientation of the molecules in thicker samples. AFM investigations were carried out with Quesant Nomad atomic force microscope in tapping mode with super-sharp silicon probes of force constant 5 N/m. All AFM scans were performed at room temperature.
